# Pediatric traumatic brain injury and early age multiple sclerosis in Finland: A nationwide register‐based cohort study

**DOI:** 10.1002/brb3.3478

**Published:** 2024-04-15

**Authors:** Juho Laaksonen, Ville Ponkilainen, Ilari Kuitunen, Julius Möttönen, Ville M. Mattila

**Affiliations:** ^1^ School of Medicine University of Tampere Tampere Finland; ^2^ Department of Surgery Hospital Nova of Central Finland Jyväskylä Finland; ^3^ Institute of Clinical Medicine and Department of Pediatrics University of Eastern Finland Kuopio Finland; ^4^ Department of Pediatrics Kuopio University Hospital Kuopio Finland; ^5^ Department of Orthopedics and Traumatology Tampere University Hospital Tampere Finland; ^6^ Coxa Hospital for Joint Replacement Tampere Finland

**Keywords:** multiple sclerosis, pediatric, traumatic brain injury

## Abstract

**Objective:**

Examine the link between pediatric traumatic brain injury (pTBI) and early‐onset multiple sclerosis in Finland.

**Methods:**

Conducted nationwide register study (1998−2018) with 28,750 pTBI patients (< 18) and 38,399 pediatric references with extremity fractures. Multiple sclerosis diagnoses from Finnish Social Insurance Institution. Employed Kaplan−Meier and multivariable Cox regression for probability assessment, results presented with 95% CI.

**Results:**

Of 66 post‐traumatic multiple sclerosis cases, 30 (0.10%) had pTBI, and 36 (0.09%) were in the reference group. Cumulative incidence rates (CIR) in the first 10 years were 46.5 per 100,000 (pTBI) and 33.1 per 100,000 (reference). Hazard ratio (HR) for pTBI was 1.10 (95% CI: 0.56−1.48).Stratified by gender, women's CIR was 197.9 per 100,000 (pTBI) and 167.0 per 100,000 (reference) after 15 years. For men, CIR was 44.6 per 100,000 (pTBI) and 34.7 per 100,000 (reference). In the initial 3 years, HR for female pTBI was 1.75 (95% CI: 0.05−6.32), and between years 3 and 20, it was 1.08 (95% CI: 0.51−1.67). For male patients, HR was 1.74 (95% CI: 0.69−4.39).

**Significance:**

We did not find evidence of an association between pTBI and early‐onset multiple sclerosis 20 years post‐initial trauma.

## Graphical abstract

There is no evidence of an association between pediatric traumatic brain injuty (pTBI) and early‐onset multiple sclerosis 20 years post‐initial trauma.Hazard ratio (HR) for pTBI was 1.10 (95% CI: 0.56‐1.48).

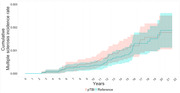

## 1 INTRODUCTION

Several neurodegenerative diseases, such as early Alzheimer's, amyotrophic lateral sclerosis, Parkinson's, and epilepsy, are often related to traumatic brain injury (TBI) as post‐traumatic outcomes. (Gardner & Yaffe, 2015; Mariajoseph et al., 2022). TBI is speculated to promote a long‐term secondary injury cascade that is caused by neuroinflammation, oxidative stress, and endoplasmic reticulum stress and leads to neurodegeneration (Kalra et al., 2022).

While pediatric traumatic brain injury (pTBI) incidence in Finland has been extensively studied in the past decade, there is a lack of research on long‐term neurological issues post‐TBI (Wilson et al., 2017; Winqvist et al., 2007). In a Finnish cohort study, pTBI incidence rose by 118% over 20 years, increasing from 251 per 100,000 in 1998 to 547 per 100,000 in 2018 (Kuitunen et al., 2023).

Multiple sclerosis is a complex disease with an unknown cause. Genes, environmental factors (like Epstein−Barr virus, Ultraviolet radiation), and central nervous system traumas could be risk factors (Milo & Kahana, 2010). There are few studies on the relation of pTBI and later‐age multiple sclerosis. A meta‐analysis of 21 case−control studies found a significant relation between head trauma under age 20 and multiple sclerosis (Lunny et al., 2014). Concussion in adolescence was also associated with an increased risk of multiple sclerosis in a Swedish nationwide register‐based study (Montgomery et al., 2017) and also in a Canadian regional retrospective cohort study that studied only Ontario province (Povolo et al., 2021). All three referenced studies showed an approximate 1.2 times greater risk of developing multiple sclerosis in patients with pTBI.

The causal relation of pTBI and early‐onset multiple sclerosis is a rarely explored topic and the subject is never studied in the Finnish population before. Therefore, we aim to examine how pTBI associates/correlates with the occurrence of multiple sclerosis at an early age and whether the pTBI patients’ gender affected the multiple sclerosis incidence.

## 2 MATERIALS AND METHODS

Two national registers named the Finnish Care Register for Health Care and the Finnish Social Insurance Institution were used to collect the data for this nationwide retrospective register‐based cohort study. The study period was from January 1998 to December 2018.

The primary study cohort included individuals with pTBI under 18, transitioning to legal adulthood (at 18 according to Finnish law) during the study period. According to existing literature, multiple sclerosis primarily affects young adults and childhood multiple sclerosis is a rare occurrence and, therefore, only patients who reached adulthood during the study period were selected (Alroughani & Boyko, 2018; Brenton et al., 2020). This group had a higher prognosis for having a diagnosis of multiple sclerosis. However, it is important to note that this manuscript did not exclude patients who were diagnosed with multiple sclerosis under the age of 18. They were patients admitted to specialized healthcare facilities for TBIs classified under ICD‐10 codes starting with “S06*.” Data came from the Finnish Care Register for Health Care, managed by the Finnish Institute of Health and Welfare (THL). This register covers patient data from secondary and tertiary‐level specialized healthcare visits, surgical procedures, and hospitalizations throughout Finland.

The reference group comprised patients under 18 at the time of the injury, transitioned to legal adulthood, and who had experienced either an ankle (ICD‐10 S82.5 and S82.6) or wrist fracture (ICD‐10 S62.5 and S62.6) requiring hospitalization between January 1998 and December 2018. This choice was based on their similar behavioral profiles and leisure activities to children with TBI. Thus, the risk of physical injury in these groups was considered comparable.

The gathered data included hospitalization dates for TBI or distal extremity fracture, along with age at injury and gender. Details on mortality and emigration during the study period were also recorded. TBI cases were categorized into operative and nonoperative treatment groups. The study determined the incidence of neurosurgical procedures using specific NOMESCO Classification of Surgical Procedures (NCSP) codes for Finland. The total surgical procedures count was obtained by analyzing the pertinent operation codes.

Excluded from the study were individuals previously diagnosed with multiple sclerosis, those who moved abroad, or those who passed away before experiencing a TBI or fracture. However, individuals who had both a TBI and a fracture were included in the TBI group for analysis.

Multiple sclerosis diagnoses, including total number and onset, were coded using special reimbursement codes (109, 157, 164, and 303) from the Finnish Social Insurance Institution. These codes cover all medications and treatment options for managing multiple sclerosis and require a comprehensive neurological examination by a specialized healthcare unit and diagnosis by a pediatric or adult neurologist. Since all Finnish multiple sclerosis patients receive reimbursement for treatment expenses, our study included all diagnosed patients in Finland.

### 2.1 Statistical analysis

Descriptive statistics included counts (%), and median with interquartile range (IQR) based on distribution. For cases of repeated hospitalizations for the same injury, only the initial one was analyzed.

Kaplan−Meier (KM) survival analysis assessed the probability of developing multiple sclerosis, with 95% CI, in both pTBI and reference groups, starting from hospitalization to multiple sclerosis diagnosis. Results were presented as cumulative incidence rates.

**FIGURE A1:**
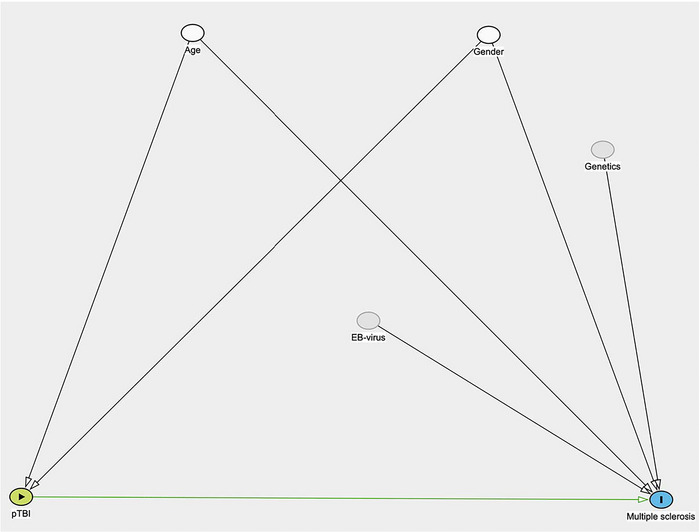
Directed Acyclic Graph (DAG) illustrating the causal relationships underpinning the multivariable model examining the impact of pTBI on the occurrence of multiple sclerosis.

Multivariable Cox proportional hazard (PH) regression analyzed cumulative incidence differences of multiple sclerosis between groups, adjusting for potential confounding factors. Covariate selection for the regression analysis was guided by a directed acyclic graph (DAG) (Shrier & Platt, 2008) outlined in Appendix 1 (Figure A1). Cox regression outcomes were demonstrated as hazard ratios (HR) with 95% CI, with multiple sclerosis as the dependent variable. Analyses were adjusted for age at hospitalization and gender.

Potential violations of the PH assumptions were scrutinized by examining the correlation between scaled Schoenfeld residuals and time. Additionally, visual assessments of the correlation between scaled Schoenfeld residuals and log‐log survival curves were conducted to assess the PH assumptions (Keskimaki et al., 2019).

Subgroup survival analyses were conducted to explore cumulative multiple sclerosis incidence differences between males and females in reference and pTBI groups. This included KM survival analysis and multivariable Cox PH regression, similar to the main analysis. Multiple sclerosis was the primary dependent variable, with adjustments for age at hospitalization and gender.

In order to address violations of the PH assumption, a time‐stratified model (Kuitunen et al., 2021) was constructed. This approach involved stratifying the study population into two intervals: 0−3 years of surveillance, and 3−20 years of surveillance. This was implemented using a time‐dependent coefficients method to correct nonproportionality.

All analyses and figures were performed using Windows R version 4.0.5 (R Foundation for Statistical Computing, Vienna, Austria) with the packages tidyverse, ggfortify, survival, and survminer.

### 2.2 Ethics

Due to the retrospective nature of the study, ethical committee approval was not needed under Finnish guidance. Data underwent pseudonymization per the personal data act (10§) by Statistics Finland. The authors did not directly access this pseudonymized data. It was handled in a secure remote‐controlled environment, requiring two‐phase identification for logins.

Permission to access the Care Register was granted by the Finnish data authority Findata, under permission number THL/4397/14.06.00/2022. Additionally, Statistics Finland permitted access to Population Information and Register of Death Causes, under permission TK/110/09.01.01/2020. Due to patient information legislation, our data cannot be publicly released. Furthermore, non‐Finland‐based researchers cannot access Finnish register data. This study aligns with ethical principles in the Declaration of Helsinki.

## 3 RESULTS

**FIGURE 1:**
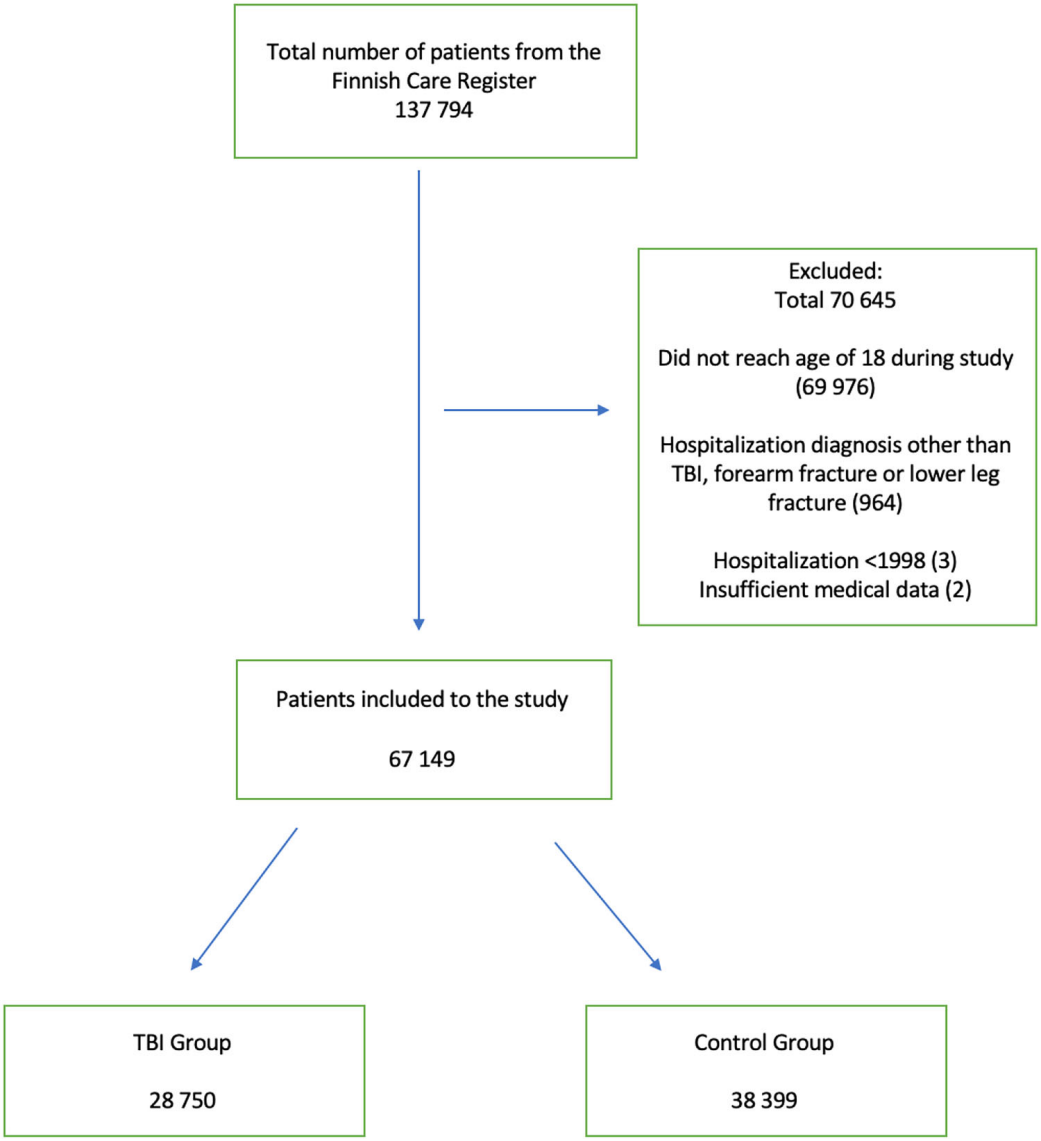
Flowchart of the study population.

From the Finnish Care Register for Health Care and the Finnish Social Insurance Institution, data for 137,794 patients were identified. After exclusions, the final study group comprised 67,149 patients (Figure 1). The primary group had 28,750 TBI patients and 38,399 references. A total of 4012 patients experienced both TBI and distal extremity fractures.

By the end of the study period, 72 patients received a multiple sclerosis diagnosis. Among them, 67 were diagnosed after TBI or distal extremity fracture, and 66 reached 18 years of age during the study. Out of these 66, 30 (0.10%) were in the TBI group and 36 (0.09%) in the reference group. Only seven patients had been diagnosed with multiple sclerosis under the age of 18. The age distribution of multiple sclerosis patients is in Appendix 1 (Table A1). Throughout the study, 648 patients passed away, and 2 out of 2375 emigrants developed multiple sclerosis. Median age at injury hospitalization was 12 years in both TBI and reference groups. In the pTBI group, 60% were males, while in the reference group, it was 66%. Among the pTBI patients, 211 underwent operative management, but none developed multiple sclerosis (Table 1).

**TABLE 1:** Median hospitalization, age, and gender distribution in pTBI‐related multiple sclerosis in Finland between 1998 and 2018.

	TBI group	Reference group
Number of patients	28,750	38,399
Median age at the time of trauma in years (IQR)	12 (8−15)	12 (10−15)
Gender, *n* (%)		
Male	17,187 (62%)	25,183 (66%)
Female	11,563 (40%)	13,206 (34%)
Multiple sclerosis, *n* (%)	30 (0.10%)	36 (0.9%)
Male	9 (30%)	
Female	21 (70%)	

Abbreviations: CI, confidence interval; ***n*, number;** TBI, traumatic brain injury.

**FIGURE 2:**
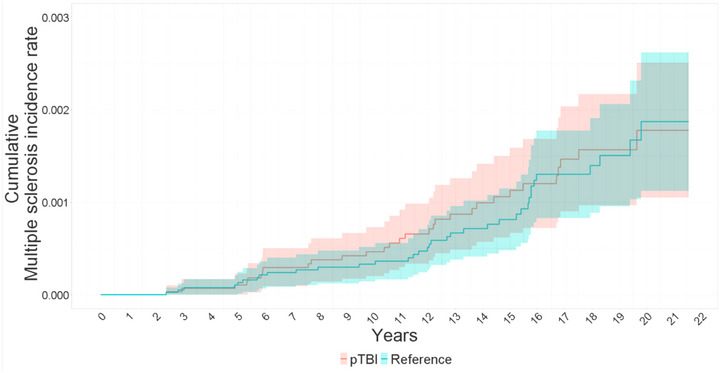
Kaplan−Meier curve of the cumulative multiple sclerosis rate in the study population with 95% confidence intervals of pTBI in Finland between 1998 and 2018.

Cumulative incidence rates after 15 years of surveillance were 112.9 per 100,000 in the pTBI group and 81.4 per 100,000 in the reference group (Figure 2 and Table 2).

**TABLE 2:** Kaplan−Meier table of cumulative incidence per 100,000 of multiple sclerosis at 1−15 years after pediatric trauma in TBI and distal extremity fracture groups in a Finnish Nationwide sample.

		at 1 year		at 5 years			at 10 years		at 15 years	
	N. of	N. risk	Cumulative incidence (CI)	N. risk	Cumulative incidence (CI)		N. risk	Cumulative incidence (CI)	N. risk	Cumulative incidence (CI)
TBI	28,750	28,619	0 (0−0)	27,205	10.7 (0−22.8)		21,753	46.5 (20.1−72.9)	14,377	112.9 (66.7−159)
Reference	38,399	38,356	0 (0−0)	37,278	10.5 (0.2−20.9)		30,529	33.1 (14.3−51.8)	18,424	81.4 (48.1−114.7)

Abbreviations: CI, confidence interval; N. of, number of patients; N. risk, number at risk; TBI, traumatic brain injury.

Based on Cox regression analysis, we did not find evidence of an increased risk of early age multiple sclerosis in pTBI patients (HR 1.10 [CI: 0.56−1.48]) compared to distal extremity patients during the 20 years study period. This result has some imprecision due to wide confidence intervals.

Subgroup analysis compared gender differences in multiple sclerosis occurrence between pTBI and reference groups.

Out of 42,380 male patients, 17,187 were pTBI cases and 25,193 had distal extremity fractures. For females, there were 24,769 in total, with 11,563 in the pTBI group and 13,206 in the reference group. Multiple sclerosis was diagnosed in 9 boys and 21 girls in the pTBI group. Median age at TBI for boys was 12 years, and for girls, it was 13 years (Table 1).

**FIGURE 3:**
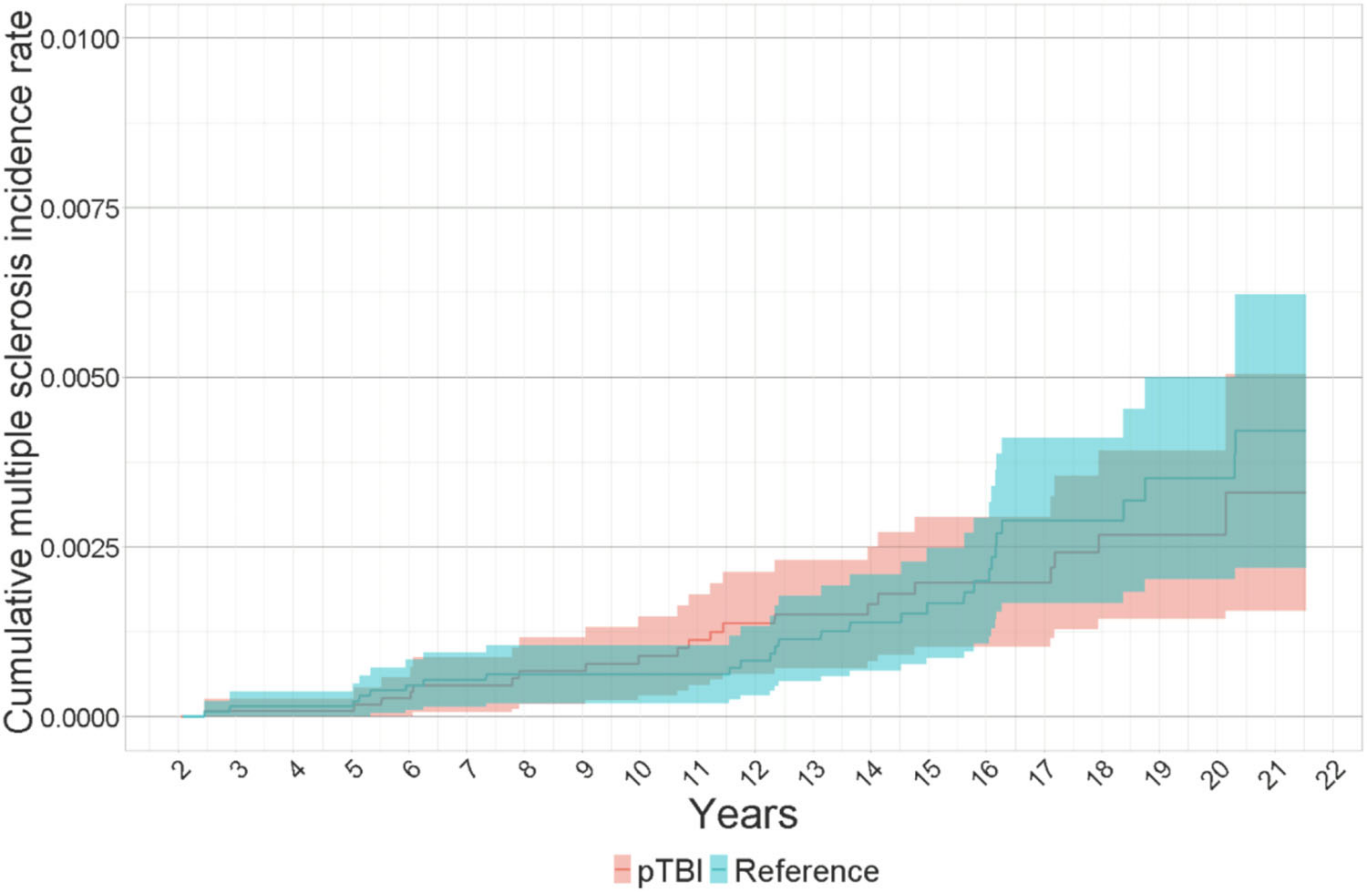
Kaplan−Meier curve of the cumulative multiple sclerosis incidence rate per 100,000 between pTBI and distal extremity fracture female patients with 95% confidence intervals in Finland from 1998 to 2018.

**FIGURE 4:**
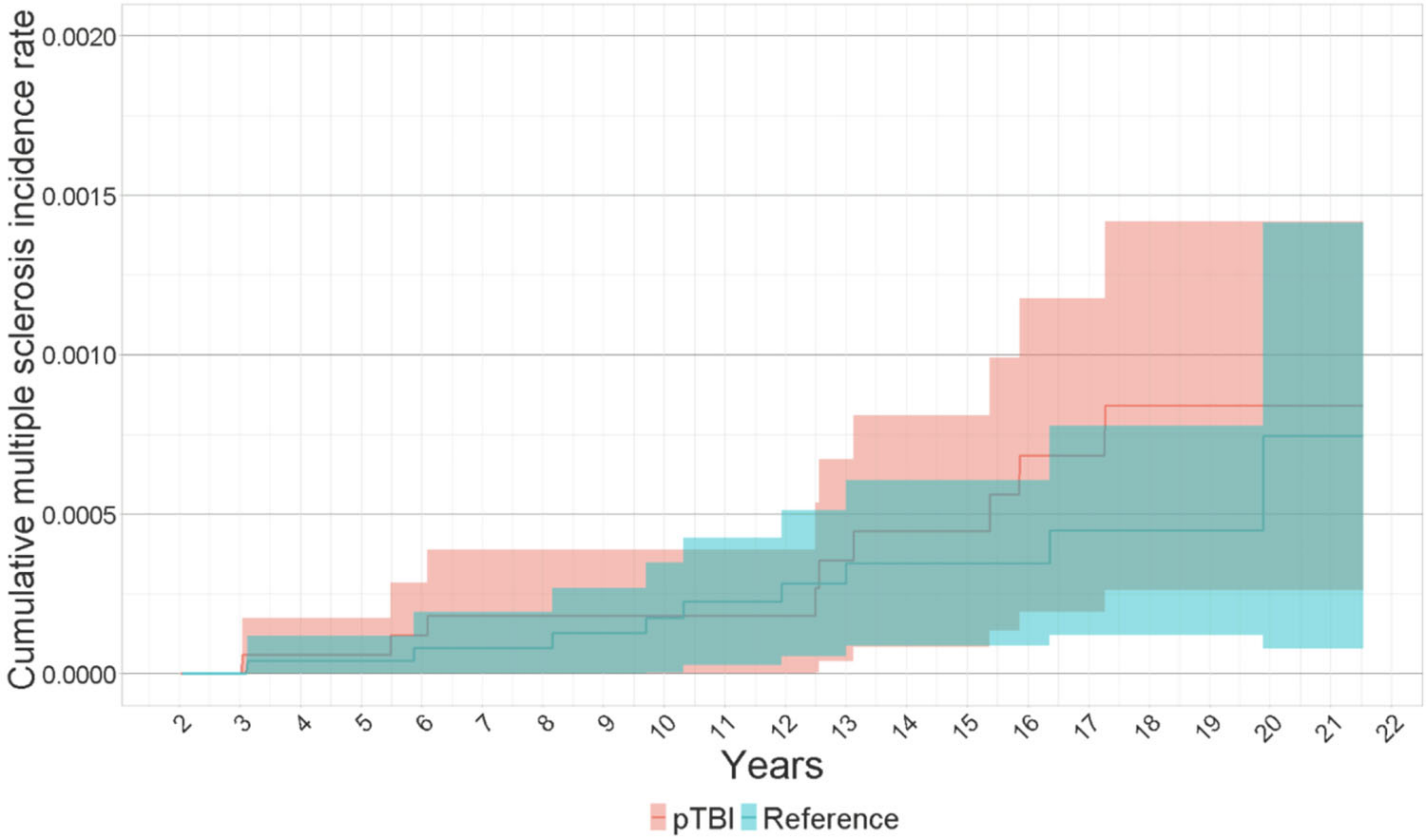
Kaplan−Meier curve of the cumulative multiple sclerosis incidence rate per 100,000 between pTBI and distal extremity fracture male patients with 95% confidence intervals in Finland from 1998 to 2018.

Cumulative incidence rates for men were 0.0 per 100,000 in the TBI and reference groups during the first 3 years of surveillance. In women, the corresponding figures were 8.7 per 100,000 in the pTBI group and 15.2 per 100,000 in the reference group. However, for women, the cumulative incidence of multiple sclerosis increased to 89.1 per 100,000 in the pTBI group and 62.1 per 100,000 in the reference group after 10 years of surveillance. After 15 years, it was 197.9 per 100,000 in the pTBI group and 167.0 per 100,000 in the reference group. For men, the figures were 18.2 per 100,000 in the pTBI group and 17.5 per 100,000 in the reference group after 10 years, and 44.6 per 100,000 in the pTBI group and 34.7 per 100,000 in the reference group after 15 years of surveillance (Figures 3 and 4 and Tables 3 and 4).

**TABLE 3:** Kaplan−Meier table of cumulative incidence rate in 1 per 100,000 of multiple sclerosis at female patients.

		at 1 year		at 3 years			at 10 years		at 15 years	
	N. of	N. risk	Cumulative incidence (CI)	N. risk	Cumulative incidence (CI)		N. risk	Cumulative incidence (CI)	N. risk	Cumulative incidence (CI)
TBI	11,563	11,563	0 (0−0)	11,466	8.7 (0−25.7)		8736	89.1 (30.7−147.5)	5936	197.9 (102.1−293.6)
Reference	13,206	13,206	0 (0−0)	13,158	15.2 (0−36.2)		11,092	62.1 (19.1−105.2)	6785	167.0 (85.6−248.3)

Abbreviations: CI, confidence interval; N. of, number of patients; N. risk, number at risk; TBI, traumatic brain injury.

**TABLE 4:** Kaplan−Meier table of cumulative incidence of multiple sclerosis rate in 1 per 100,000 at male patients.

		at 1 year		at 3 years			at 10 years		at 15 years	
	N. of	N. risk	Cumulative incidence (CI)	N. risk	Cumulative incidence (CI)		N. risk	Cumulative incidence (CI)	N. risk	Cumulative incidence (CI)
TBI	17,187	17,187	0 (0−0)	17,078	0 (0−0)		13,612	18.2 (0−38.8)	9081	44.6 (8.3−80.9)
Reference	25,193	25,193	0 (0−0)	25,109	0 (0−0)		20,096	17.5 (0.3−34.7)	12,451	34.7 (8.7−60.7)

Abbreviations: CI, confidence interval; N. of, number of patients; N. risk, number at risk; TBI, traumatic brain injury.

In subgroup analysis, we did not find evidence of increased risk for multiple sclerosis due to wide confidence intervals. During the first 3 years of surveillance in females, HR was 1.75 (CI: 0.05−6.32) and 1.08 (CI: 0.51−1.67) between years 3 and 20. In male patients, HR was 1.74 (CI: 0.69−4.39).

## 4 DISCUSSION

The study results suggest that pTBI is not associated with an elevated risk of developing multiple sclerosis compared to the distal extremity fracture group. While there is a slightly increased risk of 1.1 times, it is important to note that this result comes with some level of imprecision due to the wide confidence interval. When examining male pTBI and reference groups, as well as female pTBI and reference groups, no notable increased risk of multiple sclerosis was observed.

The previous literature on pTBI‐related multiple sclerosis is sparse. Compared to the few other previously published studies, our findings did not depict as pronounced relation between pTBI and multiple sclerosis than a large meta‐analysis of 40 studies concerning trauma‐related multiple sclerosis. This meta‐analysis had eight studies of pTBI‐related multiple sclerosis and it demonstrated that TBI increases the risk of later age multiple sclerosis OR = 1.26 (CI:1.12−1.42) and constructed for nearly 3700 multiple sclerosis patients. The same meta‐analysis had 21 premorbid TBI studies that demonstrated an increased risk of multiple sclerosis after TBI regardless of patients’ age (OR = 1.65 [CI: 1.39−1.95]). (Lunny et al., 2014) The meta‐analysis noted a significant issue in the studies included: they either lacked clear indications of TBI severity or employed varying criteria for assessment. This inconsistency in severity assessment raises concerns about potential biases in the results.

Additionally, the association between pTBI and multiple sclerosis varied depending on the study design. Prospective studies showed the weakest association (OR = 0.94 with CI: 0.77−1.15), while case−control studies exhibited the strongest (OR = 1.65 with CI: 1.39−1.95). This suggests that the choice of study design, especially the usage of case−control studies, may introduce bias due to clinical diversity among patients.

A large Swedish case−control study of head trauma‐associated multiple sclerosis in adolescence (before the age of 20) consisted of over 7000 multiple sclerosis patients. As a result, they found a significant correlation between concussion in adolescence (age 11−20) and later age multiple sclerosis aOR = 1.22 (CI: 1.05−1.42) after one head injury and the risk was over twice (aOR = 2.33 [CI: 1.35−4.02]) if the patient had more than two head injuries. However, they did not find a correlation between childhood (under the age of 10) TBI and later age multiple sclerosis. Swedish cohort could be considered similar to our population since both countries are located in Fennoscandia and have similar socioeconomic infrastructure. However, the Swedish study had a larger population of multiple sclerosis patients due to different study types of case−control and a much longer surveillance time from 1964 to 2012. Swedes also divided the study population into children and adolescents and found a correlation only with adolescent concussion. (Montgomery et al., 2017) A Canadian study of nearly 100,000 pTBI patients had similar findings to Swedes. They found out that TBI in adolescence (age 11−18) increases the risk of multiple sclerosis (HR = 1.29), but unlike our results, they demonstrated that the risk was higher among males (HR = 1.41), whereas our study demonstrated over 1.5 times higher risk for women (Povolo et al., 2021). The Canadian study was also similar in study design to ours and they studied retrospectively TBI patient cohort. However, they included patients only with concussion.

Our incidence of multiple sclerosis correlated with an earlier Finnish study which found out that the 2012−2016 incidence of multiple sclerosis in all ages, in two Finnish regions, was 10.7/100,000 person‐years (CI: 9.4−12.0) (Zhang et al., 2018).

Our study has several strengths. Of these, the main strength is the exceptional quality of the registers incorporated (Huttunen et al., 2014; Pirttisalo et al., 2019; Sund, 2012). ICD‐10 classification has been used in Finland since 1998 and the coding measures have remained similar throughout the study period. Furthermore, prior research on neurological conditions has successfully utilized the Finnish Care Register for Health Care, employing ICD‐10 coding in tandem with data from the Finnish Social Insurance Institution (Kallionpää et al., 2021; Mattila et al., 2008). This underscores the credibility of our chosen data sources. Another strength is the free specialized healthcare visits for children in Finland. The social insurance system, funded by universal fees based on individual income, ensures that healthcare expenses are covered (Feuth et al., 2020). This equitable provision of healthcare minimizes potential socioeconomic biases. Both the high‐quality registers and the reimbursement system are provided by the Finnish national healthcare system and serve to enable qualified register studies. Additionally, the robustness of our study is bolstered by the expansive population of over 70,000 patients with pTBI. Furthermore, the limited variation in sex and age between study groups helps minimize potential sources of bias, strengthening the validity of our results. A final strength of the study was the use of special reimbursement codes collected from the Finnish Social Insurance Institution. The strict criteria for medication reimbursement, contingent on confirmation by a pediatric neurologist, ensures the precision and reliability of multiple sclerosis diagnoses in our study population.

Our study has some limitations. First, the Finnish Care Register for Health Care does not include data on patients’ familial medical history. Consequently, we could not assess the impact of genetic factors on multiple sclerosis. Given that multiple sclerosis is influenced by both genetic and environmental elements, this is a crucial aspect to consider (Milo & Kahana, 2010). Additionally, our study's observation period was relatively short and the number of multiple sclerosis patients was limited. Existing literature underscores that multiple sclerosis primarily affects young adults, making it a rare occurrence in children. Although a small percentage of patients may experience symptoms before the age of 20, diagnosis typically occurs at a later stage (Alroughani & Boyko, 2018; Brenton et al., 2020). Another point to note is that registry data might incorporate diagnostic inaccuracies arising from the coding of pTBI diagnoses by healthcare practitioners. Third, the study data do not contain information from primary care. Since mild injuries are usually managed by primary care providers, it is probable that the incidence of less severe injuries may have been underestimated in our analysis. Fourth, the registers used in this study did not include Glasgow Coma Scale ratings nor other severity ratings, such as duration of posttraumatic amnesia nor diagnostic criteria for multiple sclerosis such as the McDonald criteria, which have evolved during the study period. Fifth, our data solely pertained to the initial TBI. Consequently, we were unable to assess the cumulative risk associated with multiple TBI injuries of varying severities and their potential connection with multiple sclerosis using our register data. Sixth, our dataset had a limited number of potential covariates, and therefore, the Cox regression model was adjusted only for age and gender. Finally, only the special reimbursement codes of the Finnish Social Insurance Institution were used for multiple sclerosis diagnoses. As a result, the severity of multiple sclerosis could not be evaluated. Information on medication purchases for multiple sclerosis was also not available, and there was no specific information of possible clinical trial patients. Another notable limitation is the absence of information regarding the specific types of multiple sclerosis in our dataset. Recognizing that different types of multiple sclerosis may respond differently to pTBI, the lack of subtype classification restricts our ability to explore potential variations in the impact of pTBI on distinct multiple sclerosis subtypes.

In the future, it would be valuable for research to investigate how various posttraumatic treatment strategies for TBI influence the incidence of multiple sclerosis in later years. This study could potentially identify treatment modalities that demonstrate superior efficacy in reducing the incidence of multiple sclerosis. Given the rarity of multiple sclerosis, particularly in pediatric cases, it is imperative that further research on TBI‐associated multiple sclerosis is conducted with larger cohorts and extended surveillance periods. This approach would significantly contribute to our understanding of this complex interaction.

## 5 CONCLUSION

The findings from this nationwide cohort study did not demonstrate an association between pTBI and elevated risk of early‐age multiple sclerosis. Furthermore, no correlation was observed when comparing males and females separately. These results highlight the significance of TBI prevention and provide information on how long‐term posttraumatic monitoring or treatment may influence the incidence of multiple sclerosis   .

**TABLE A1:** . Median age distribution in years among multiple sclerosis patients at the time of trauma, diagnosis, and at the end of surveillance.

	pTBI	Reference
Time of trauma	15 (12−15)	13 (9−15)
Time of multiple sclerosis	23 (20−28)	23 (19−28)
End of surveillance	28 (25−33)	29 (26−33)

## AUTHOR CONTRIBUTIONS

**Juho Laaksonen**: Writing—original draft; writing—review and editing; data curation. **Ville Ponkilainen**: Conceptualization; writing—review and editing. **Ilari Kuitunen**: Writing—review and editing; conceptualization. **Julius Möttönen**: Data curation; writing—review and editing. **Ville M. Mattila**: Writing—review and editing; conceptualization.

## CONFLICT OF INTEREST STATEMENT

None of the authors has any conflict of interest to disclose.

### PEER REVIEW

The peer review history for this article is available at https://publons.com/publon/10.1002/brb3.3478.

## PATIENT CONSENT STATEMENT

According to Finnish research legislation (Law on medical research and Law on the secondary use of routinely collected healthcare data), patient consent is not required when the participants in retrospective register studies are not contacted.

## Data Availability

The Finnish law on secondary use of healthcare data prohibits the public sharing of our data. However, researchers may apply for research permission from the Finnish data authority, Findata, via e‐mail to gain access to the data, e‐mail: info@findata.fi
